# Awareness and understanding of concussion among Aboriginal Australians with high health literacy

**DOI:** 10.2217/cnc-2023-0012

**Published:** 2024-04-24

**Authors:** Trish Hill-Wall, Kahlia McCausland, Elizabeth Thomas, Richard Norman, Jonathan Bullen, Gill Cowen

**Affiliations:** 1 Moorditj Yorga Scholarship Program, Curtin University, Bentley, 6102, Australia; 2 Curtin Medical School, Curtin University, Bentley, 6102, Australia; 3 Curtin School of Population Health, Curtin University, Bentley, 6102, Australia; 4 Centre for Clinical Research Excellence, Curtin University, Bentley, 6102, Australia; 5 Division of Surgery, Faculty of Health & Medical Sciences, The University of Western Australia, Perth, 6009, Australia; 6 Curtin enABle Institute, Curtin University, Bentley, 6102, Australia; 7 Curtin Health Innovation Research Institute, Curtin University, Bentley, 6102, Australia

**Keywords:** Aboriginal, concussion, First Nations, indigenous, mild traumatic brain injury, mTBI, Torres Strait Islander, traumatic brain injury

## Abstract

**Aim:**

Indigenous Australians have higher rates of traumatic brain injury, with 74–90% of such injuries being concussion. This study explores concussion awareness and knowledge in Aboriginal Western Australians with high health literacy.

**Materials & methods:**

Participants, aged 18–65 years, engaged in research topic yarning, and thematic analysis of the qualitative data then undertaken.

**Results:**

There was awareness that direct head trauma can result in concussion, but a lack of differentiation between concussion and other head injuries. Knowledge was gained from sport, media or lived-experience. Symptom minimization and diversity of concussion symptoms prevented participants from seeking medical treatment. This was exacerbated by a mistrust of the medical system.

**Conclusion:**

Research findings highlight knowledge and service gaps where co-designed strategies can be targeted.

## Plain language summary

Despite higher injury rates in Indigenous Australians, literature relating to brain injuries such as concussion in these populations is lacking. This article provides information regarding awareness and knowledge of concussion in Aboriginal peoples with health or first responder experience. Through the process of yarning, 25 Aboriginal participants shared their awareness and knowledge of concussion. While there was good understanding of how concussion injury can occur, it was found that identification of concussion as a diagnosis is complex, and it is hard to differentiate from other conditions. Minimization of concussion symptoms was commonly reported, and multiple barriers to seeking healthcare after a potential concussion occurs were identified. Despite having health or first responder experience, participants reported their concussion knowledge was gained from community and televised sport, other aspects of media, and word of mouth. These results support the need for Indigenous Australian led and co-designed concussion education. They also support the need for further research in this space, targeting Indigenous Australian populations without high health literacy.

## Tweetable abstract

Research topic yarning with Aboriginal healthcare workers and first responders about concussion has highlighted the need for Aboriginal and Torres Strait Islander peoples led, co-designed, concussion education.

Concussion is a disturbance in brain function that occurs as a result of a direct blow or transmitted force to the head. Recent data estimates there are 190,000–200,000 cases of traumatic brain injury per year in Australia [1], of which 74–90% are probable concussions [2,3]. Despite Indigenous Australians representing a higher proportion of hospitalizations for head injury compared with non-Indigenous Australian people [4], literature and data relating to concussion in these populations are scarce [5].

It is estimated that only approximately 20% of concussions are sports-related [6,7], with the majority of concussions resulting from falls, pedestrian and vehicle-related road crashes, and assaults, including intimate partner violence [8–12]. Concussion has been repeatedly identified as being under-reported in sporting populations [13–15] and while it is assumed that under-reporting of non-sports-related concussions also remains high, there is a lack of evidence to support this due to limited literature on non-sports-related concussions.

Given the higher rates of physical injury among Indigenous Australians [16], awareness and understanding relating to potential concussion injuries and access to culturally appropriate health services in the event of such injuries in these populations must be identified. The first steps in supporting equitable and positive health outcomes are determining and understanding the drivers of, and factors that may impact Indigenous Australians presenting for assessment after sustaining a potential concussion.

This study investigates awareness and understanding of concussion and the factors that may impact presentation for assessment after a potential concussion injury among Indigenous Australians with high health literacy (background in healthcare or first response) living in Perth, Western Australia. Although it is assumed that persons with a background in healthcare or first response will have undertaken concussion-related training, literature reports health professionals are not adequately educated nor confident in concussion diagnosis or management [17]. Findings from this study will provide information to inform future work looking to improve concussion education and presentation rates for concussion assessment and management in Indigenous Australians.

The terms mild traumatic brain injury (mTBI) and concussion are often used interchangeably, with mTBI encompassing concussion (no changes on routinely available neuroimaging) and complex mTBI. The term ‘concussion’ may be preferable when communicating with the lay community since it avoids the stigma associated with brain damage or injury [18] and is used in this paper to describe the occurrence of an injury that causes a transmitted force to the brain resulting in functional neurological disturbance.

## Methods

### Research team

The research team consisted of three Aboriginal Australians and three non-Aboriginal investigators. JB is a Wardandi Noongar man, whose research focuses on expanding the lens through which Aboriginal health and well-being are viewed, understood and promoted. ET is from the Yamaji region and works as a Research Fellow coordinating research across rural and remote Western Australia. TH-W is a proud Noongar Elder. GC is a rural-trained General Practitioner, RN is a Health Economist whose research focuses on health outcomes and evidence-based policy and KM has experience conducting research with marginalized populations.

### Methodology

This qualitative research study adopted a social constructionist lens, which places knowledge within the process of social interchange [19], informed by decolonizing methodologies and concepts of the cultural interface to privilege Indigenous ontologies [20]. This adoption facilitates an exploration of the meanings Indigenous Australians ascribe to their experience and enables the investigators to acknowledge the influence of their worldviews on the research process, findings, and implications derived. This study used a yarning research process called Research Topic Yarning “*to gather information through participants' stories that are related to the research topic. While the yarn is relaxed and interactive it is also purposeful with a defined beginning and end*” [21].

The reporting of this study is guided by the consolidated criteria for reporting qualitative research (COREQ) [22] checklist (Appendix 1) and the CONSolIDated critERia for strengthening the reporting of health research involving Indigenous Peoples (CONSIDER) [23] statement (Appendix 2).

### Sampling & recruitment

Eligible participants were required to identify as Aboriginal or Torres Strait Islander, be aged between 18 and 65 years and be currently staying or residing in Perth, Western Australia. Participants were recruited through purposeful sampling. TH-W has an established professional connection with a Registered Training Organization (RTO) for Indigenous Australians who agreed to support the research, assist with recruitment, and provide their facilities for data collection. The RTO posted a recruitment flyer throughout its campus and conducted in-class recruitment drives whereby a verbal overview of the research was provided and interested people could provide their contact details for follow-up. TH-W contacted potential participants and invited them to attend one of two yarning circles, where they would have the opportunity to share their ideas and experiences in a safe space. Four participants were recruited through the personal networks of TH-W (word of mouth) who chose to participate in a 1:1 yarn.

### Data collection

The research team (GC, TH-W, ET, JB) developed a yarning topic guide (Appendix 3) that identified questions relevant to the research aim. The topic guide was used to prepare the researchers for data collection rather than directly determining the path of questioning. The topic guide was referred to during yarns as a set of prompts to ensure the identified areas of interest were discussed. Additional prompts were used as appropriate to elicit participants' stories and experiences that came about during the natural exchange of information.

Yarning circles were conducted at a culturally secure space (RTO campus) and via 1:1 yarns at a mutually convenient neutral location (e.g., open outdoor space). Yarns were scheduled at a suitable time according to community protocols (e.g., avoiding cultural ceremonies or sorry business) and participant availability.

All of the 1:1 yarns were facilitated by Aboriginal researcher TH-W. The first yarning circle was facilitated by TH-W and supported by non-Aboriginal researcher GC. During this yarning circle, GC observed how the session was facilitated, took notes and when appropriate offered further prompts. GC then went on to facilitate the second yarning circle. Before the second yarning circle, TH-W provided GC with information on the unique Aboriginal groupings attending the session and the appropriate protocols to use before, during and after the yarn.

At each of the data collection activities, TH-W was present to meet and greet the participants, provide a verbal overview of the research and engage in a social yarn [21]. During the social yarn, the researcher/s provided participant/s with personal information about themselves, their interest in the project and their reasons for being involved, and provided participants with the opportunity to reciprocate. This process facilitated mutual respect and trust (also imparting richer insights and deeper yarning [24]), particularly if no prior relationship was established between the researcher/s and the participant/s. TH-W had an established relationship (professional and/or moort (family) with some participants which further created a favorable and safe space. At the close of the social yarn, TH-W, with the aid of a participant information sheet, provided participants with verbal information about confidentiality and privacy, the audio recording, transcription and analysis process and the proposed nature of the discussion. Participants were provided time to consider the information provided to them and to ask questions before providing written consent to participate. Consent to audio record the yarn (lasting between 1 and 2 h in duration) was obtained from every participant. Participants were made aware that they were able to withdraw from the study at any time and given the nature of the topic under investigation participants were able to remove themselves from the yarn at any time for a break. Participants were offered post-yarn support through Yorgum Healing Service.

Following the provision of participant's consent, they were asked to complete a simple demographic survey (Appendix 4) before moving into the research yarn whereby participants were encouraged to share their (and their mobs’) experience of potential concussion (including how these situations were/were not managed, experiences of medical assessment and recovery journey), and their awareness and understanding of the term concussion. During the exchange of knowledge between the researcher/s and participant/s, incidental concussion education was also provided.

As a token of appreciation, refreshments were provided and a $50 gift card for a supermarket chain (Coles-Myer) was offered to participants for their time, contribution, and willingness to share their experiences and knowledge.

### Data analysis

Audio recordings were transcribed by a professional transcription service and then reviewed for accuracy (KM) to facilitate data familiarization and the generation of initial codes. In instances where the Aboriginal language was used and not recognized JB provided clarification. Anonymized transcripts were imported into NVivo [25] to facilitate data organization and linkage. Reflexive thematic analysis, the six-phase process set out by Braun and Clarke [26] was used to engage with the data. Two authors (GC, KM) inductively coded all transcripts to generate codes relevant to the research question. Once all transcripts were coded the authors came together to discuss the codes they had developed. This process was helpful for KM (who has no prior knowledge of concussion) to better understand the pathophysiology, symptoms and signs of concussion. KM was then able to refine the codes based on her better understanding and the appropriate use of concussion-related language. Connected codes were then collated under potential themes. At this point, a preliminary analysis report with supporting participant extracts was drafted and reviewed by the broader research team. Subsequently, another round of analysis (level 2, Appendix 5) occurred whereby themes, codes and the accompanying narrative were revised and again reviewed by the broader research team. A final round of analysis (level 3, Appendix 5) was conducted to refine themes and sub-themes, the associated narrative summary, and the overall narrative. Transcripts and summary preliminary analysis were not returned to participants for comment, nor were follow-up yarns carried out. Demographical data were aggregated in Excel and analyzed using descriptive statistics.

### Ethical considerations

Before applying for ethics approval, a request for project support was submitted to and approved by Derbarl Yerrigan Health Service (DYHS) Aboriginal Corporation Research and Development Sub-Committee. DYHS' process for reviewing research proposals has been designed in consultation with the Western Australia Aboriginal Health Ethics Committee (WAAHEC) and appraises projects using the Guidelines for Ethical Research in Australian Indigenous Studies [27]. Ethics approval was obtained from the WAAHEC (HREC1012) and reciprocal ethics approval from Curtin University (HRE2020-0690).

## Results

Twenty-five participants were recruited; 21 through the RTO and 4 via word of mouth. Twenty (80%) participants identified as females, 4 (16%) as males and one as other gender (Table 1). All participants recruited from the RTO were undertaking further education (Certificate of Primary HealthCare). The other four participants were a registered nurse, medical school lecturer, police officer and community support worker in aged care. All participants identified as Aboriginal.

**Table 1.** Participant demographic characteristics.

Characteristic	n	%
Age		
<25 years	6	24
25–45 years	9	36
>45 years	10	40
Gender identity		
Male	4	16
Female	20	80
Other	1	4
Education		
Year 10	22	88
Year 12	7	28
Vocational education and training (VET)	15	60
Higher education	1	4
Student		
Yes	7	28
Employed		
Yes	9	36
Carers duties (immediate/extended family)		
Yes	9	36

## Findings

Five predominant themes were identified. A narrative summary, with supporting quotes, is presented. Quotes have been attributed a pseudonym to protect the participant's identity. In the case of yarning circle participants, it was not possible to attribute the participant's age.

### A good understanding of how concussion injury can occur

Participants reported having seen sporting players knocked to the ground and the word ‘concussion’ being associated with such an event. The most frequently identified mode of concussion injury was sport, predominantly Australian rules football, rugby, boxing and netball.

Maisie (F, 25–45): *“I just associate those two words together. Boxing and concussion.”*

Maisie (F, 25–45): *“… It's generally in a sporting sort of scenario. … Nowhere else really.”*

Bobby (M, >45): *“*… *you see a fella cop a whack now … and even in the cricket, you know, test cricket …”*

Experiences of family members and intimate partners sustaining a concussion injury secondary to a heavy blow to the head during sport were common. There was also evidence of knowledge that falls, interpersonal violence and road traffic incidents can result in a concussion.

Jim (M): *“Well, if you're assaulted and you fall or you hit your head, or even if you were in a car accident …”*

Some participants specifically referred to mourning in traditional Aboriginal culture as a possible way a concussion could be sustained.

Margaret (F): *“How are you going to tackle the traditional mob that hit themselves when there's a death in a family?”*

Facilitator: *“When there's funeral, it's part of funeral.”*

Libby (F): *“Mmm, sorry business.”*

Margaret (F): *“That's a big one.”*

The impact of alcohol and other drugs within Aboriginal communities was also recognized as increasing concussion injury risk.

Jim (M): *If they [inebriated person] don't know what they're doing, they're going to get hit that hard by a bloke who's sober. Now you see a lot of, not only Noongars*^1^ *but Wagala's*^2^ *too once they get a tank full [of alcohol] they want to fight.*

Margaret (F): *“And they go looking for a hidin'.”*

Jim (M): *“If you work up in these communities … where these traditional people fight. I mean, they're smashing each other over the head with big nulla nulla [wooden club]. So it's always, they're getting concussion …”*

### Concussion as a diagnosis is complex & it can be difficult differentiating it from other conditions

Most participants were unaware of the fact that a concussion can be sustained from a transmitted force because of a blow to the body, with participants identifying a blow to the head as the primary mechanism for a concussion. No participant provided information relating to the underlying pathophysiology of concussion and only one provided information relating to an understanding of what occurred in the brain when a person sustained a concussion.

Jim (M): *“It [concussion] is, uh, bruising on the brain.”*

Facilitator: *Can you tell me a bit more about what you mean by bruising on the brain?”*

Jim (M): *“*… *because your brain is only sitting in the centre, and it is rockin' around.”*

Participants expressed that it is difficult to determine if someone is suffering from a concussion, especially if there is no visible indication of head injury, or if the incident was not witnessed.

Tilly (F, >45): *“… so I think this is where the health literacy … of the people around them is really important because it's actually noticing, um, it [concussion] can be subtle, but, subtle signs.”*

Furthermore, some participants commented that people who use alcohol and other drugs display similar symptoms and signs to someone who may be suffering from a concussion, making it difficult to determine whether the person is intoxicated, concussed or both.

Tilly (F, >45): *“How can you tell the difference between um, having a hangover and actually showing signs of concussion?”*

Participants had difficulty differentiating between the symptoms and signs that constitute a concussion rather than an alternative head injury diagnosis. While able to identify symptoms and signs that are red flags for a head injury, they were unable to differentiate between presentations suggestive of concussion and those which suggest an alternative diagnosis. The term concussion was used as a highly inclusive term relating to a blow to the head in multiple scenarios where information provided suggested an alternative traumatic brain injury or another diagnosis.

Jessy (F): *“I got on top of a motorbike and got fairly well-concussed.”*

Facilitator: *So, what do you remember about that? …“*

Jessy (F): *“Well, I remembered a lot of pain, and I remember a lot of vomiting. … I ended up in a coma. … I was in coma for about 15 hours. Um, and then, obviously, I had an operation with my head. … That took months. Three months, at least [to recover].”*

The predominant sign of a concussion identified by participants was the loss of consciousness or ‘blacking out’. For example, some participants recalled seeing a rugby player on TV who had been ‘knocked out’ and they described the player's subsequent behaviour.

Margaret (F): *“And then he was just laying there. And you could tell that he was not orientated to time or place.”*

Donna (F): *“He wasn't. He was stiff.”*

Margaret (F): *“They were talking to him. He's like looking around and didn't, he was dazed and confused.”*

Donna (F): *“He couldn't move.”*

Margaret (F): *“They had to get the stretcher and ambulance.”*

Donna (F): *“Couldn't even walk!”*

Sleepiness, slurred speech, blurred vision and dilated pupils were also reported by participants as possible signs suggesting concussion. Vomiting was frequently referenced as a symptom of concussion and other symptoms included being disorientated, confused or ‘dazed’, memory loss, difficulty concentrating, fatigue, headache, dizziness, ‘wobbly legs’ and problems with balance including ‘staggering’ and ‘dragging legs’. A jarred or sore neck was also reported as a possible concussion identifier.

Participants who had experienced a concussion identified headaches as a symptom of concussion. Headaches were described as dull in nature, severe in intensity and lasting between hours and days.

Bobby (M, >45): *“You do suffer with headaches. I've had headaches for days after [a knock to the head]. … I've had them [headache] for, you know, over an hour at times. With it, I go into a dark room. … I try to sleep things off.”*

Other symptoms identified as associated with concussion included low mood and heightened emotions. Those participants who identified anxiety as a symptom also referenced changes in behavior such as aggression, and fluctuations in mood.

Bobby (M, >45): *“Some people can get real loopy, you know, and go right off. Become very aggressive. Yeah, there's a change in behaviour.”*

### Concussion awareness & knowledge is acquired via sport, media & lived experience

Participants shared the perception that there is as of lack knowledge and awareness of concussion:

Maisie (F, 25–45): *“I think, um, concussion's not really widely spoken about in- I mean, I wouldn't know in the sporting world anymore. I'm not sort of predominantly in it as much as I used to be, but I know that it [concussion] wasn't spoken about at all, wasn't even talked about, thought of, whatever. But nowadays, I think, it is spoken about, but I don't think there's still enough information getting out, especially to the younger generation. They're very, um, impressionable. You know, they want to be the best, be the toughest – you know, at the expense of possibly hurting themselves.”*

Concussion knowledge was typically acquired by witnessing how potential concussions were managed at community and televised sporting events. Further knowledge and awareness came from the media and through the passing of intergenerational knowledge and personal experiences of concussion.

Maisie (F, 25–45): *“Um, concussion, probably just on TV, in papers, magazines, hearing about other people's stories.”*

Bobby (M, >45): *”*… *in the cricket, you know, test cricket. And, uh, they stop the game; medical people come out; they check the helmet, the safety equipment. They do their little protocol tests and that sort of thing."*

Tilly (F, >45): *“I heard about concussion because my mother was a nurse. … So, she was very mindful of signs and symptoms of concussion, even when I was growing up.”*

Media had also provided participants with an awareness of potential long-term sequelae of concussion with older participants acknowledging that they were starting to question whether some of the particular behaviours (e.g., aggression), symptoms (e.g., memory loss) and other conditions (e.g., Parkinson's disease, mental health challenges) that prominent sports people (e.g., Muhammad Ali, Polly Farmer), they, their family and friends were experiencing in later life were a result of previous head injuries.

Maisie (F, 25–45): *“… getting older now, now that I know that my mum has you know, mental health issues, can't help but think, you know, are they [head knocks and mental health issues] actually related in some kind of way?”*

### Symptom minimization is common

In adults, minimization of injury or symptoms was commonly reported.

Maisie (F, 25–45): *“I mean, my dad's a blackfella, you know … Unless you're dying and you're bleeding to death or something. That's when you went to the doctors. So if you're, if you grow up around that, you sort of tend to take on that attitude as well too. You think that's the norm, you know?”*

One participant explained concussion is perceived as ‘really not that serious’ (Pat, F) and past experiences were reported to have resulted in certain behaviors being passed on to subsequent generations:

Marj (F): *“Because all of our Elders, they used to get hit all the time. Just get bashed around. So, you know, you grow up to learn that.”*

Libby (F): *“And we don't think of the worst when it comes to that [concussion]. We never think of the worst. You don't think about dying.”*

Several older participants referred to a culture within sporting clubs when they used to play where players were taught to *have* ‘eyes only for the ball’ and to ‘aim to win above all else’. A particular *“focus on not wanting to let the team down”* (Jim, M), was highlighted by several sport-playing participants who explained that personal injury symptoms were unimportant when compared with commitment to the team.

Bobby (M, >45): *“… there's that obligation about not letting your mates down … so you keep going at all costs ay.”*

Bobby (M, >45): *“Even though I'm not a soldier or I wasn't at war or anything like that. But you commit to something [football], and you follow it through. You sacrifice because anything that's worth having, you pay a price.”*

A similar culture and commitment to the team was also reported among younger participants who recounted times when they continued to play out a game after a potentially concussive event or returned to play after being side-lined for a brief period.

Marj (F): *“This player tripped me [in netball]. Donk! Straight on the floor. And um so you know, you get a bit disorientated, trying to get up on your feet, but I kept playin'. (laughs)”*

### There are barriers to seeking healthcare after sustaining a potential concussion

Some participants explained that residue effects from colonization and Australia's history of suppression linger among many Indigenous Australians and consequently they do not prioritize their health and are reluctant to present to health services.

Libby (F): *“We'll come back to colonisation and all of those sorts of things, blackfellas been suppressed all the time. So they don't really care about their health.”*

For Indigenous Australians, particularly those from the Stolen Generation, health services (hospitals in particular) were perceived as oppressive institutions that can represent separation from family and death.

Bobby (M, >45): *“I think it's a combination of things, um, if I'm being honest. One is the understanding of it, but secondly, what hospitals represent for our mob too, you know? Because I think there's a cultural implication that we've had - traditionally, over the years, when you go to hospital, one, you either die, or they remove you. So, we're talking in a modern-day society now, in a Western way that would be thinking, ‘Well, they're stupid.’ But no, because there's still those Stolen Generation thread-lines, is what I call them - that impact on behaviours. Um, with it. So, I think for our mob, when you're talking about hospitals, you're going to die or someone's going to take ya.”*

Nell (F): *“*… *like my mum being in the Stolen Generation. It's a lot harder for her to go [to the hospital]. That's just the last place you would want to go. I can't really explain it because I've never been through it. But I can see that fear in her eyes … like, no, she's not going …”*

Other factors also impact on presentation for assessment. In one yarning circle, women participants detailed that home and carer's duties (i.e., children, extended family) have to be prioritized and this often precludes them from seeking personal medical care for a range of health issues, including concussion.

Marj (F): *“Our life … doesn't stop just because we got to take the time for ourselves. … Um, mothers and women, we don't make ourselves the first priority.”*

There were also concerns for some who played competitive sports that if they presented at a health service after sustaining a potential concussion they would be told “they can't play … anymore.” (Bobby, M, >45)

The paucity of Indigenous Australians working within health services and the effect on presentation for medical assessment was discussed by participants. Participants said they did not expect to be treated with respect, taken seriously or provided with culturally appropriate consultation and care. For example, one participant commented that many Indigenous Australians do not engage with government-mandated services due to the anticipation that they will not be adequately assisted:

Tilly (F, >45): *“… if you're gonna go up [to the hospital] and go and get tested [for concussion]. What do they [Aboriginal person] anticipate is going to be done for them? So the disappointment from actually hoping and expecting, and having not an unreasonable expectation that when you present to any government department or any department for the services that they provide. Whether they deliver- And more often than not, they don't. And so, it's a little bit like well, it's, you know, there's an understandable level of despondency and disengagement from participating in what is available out there, because will they do anything?”*

The same participant commented on the perceived disingenuity of some government-funded ‘Aboriginal’ health services whose mandate is to service the Indigenous community, however, were not seen to be doing so.

Tilly (F, >45): *“… there's a heck of a lot of organisations that are setting themselves up to be able to deliver, with the idea of delivering services to Aboriginal people, because they get funded by the Closing the Gap*^3^. *But they're not actually doing one bit of delivery to the [Aboriginal] people, because they don't actually know how to do it, who to ask, um, what to do.”*

The lack of male Indigenous health professionals was reported to further compound men's avoidance of health services. Indigenous Australian men often prefer to discuss their business man-to-man, especially for sensitive health issues such as mental health.

Jessy (F): *“And we'd like to see a few more [Aboriginal] males [working in healthcare]. I don't know what it's like down here [Perth], but there's not very many in the country.”*

Nell (F): *“There's hardly any [males] here too. And there needs to be more males, just to be culturally respective … Like, it's easier for a woman to go to a doctor because there are a lot of women doctors. But for a man, like-”*

Jessy (F): *“That's true. And even mental health, you know? They need more males in mental health.”*

There was hesitancy to visit a health service for concussion assessment when under the influence of alcohol and/or other drugs because of the perceived inadequacy of non-Indigenous health professionals who were reported as unfamiliar with how to engage with an Indigenous person who is intoxicated.

Linda (F, >45): *“And you can't take them to the doctors when they're … that drunk, the doctor doesn't know how to talk to them or treat them. And they [doctors] get a bit frightened of, um, drunken people, you know? So, um, we got to wait for them to be sober enough so that we can get them to see a doctor, but half of them don't want to. They say, no we're alright …”*

Participants also commented that they had experienced, or feared, authorities would be contacted due to prejudice and discrimination upon presentation at a health service with a concussion.

Jessy (F): *“You just think the worst, really. You know, because we are so stereotyped. You get a brain injury, ‘Oh, well, who were you fighting?’”*

Pat (F): *“Or, ‘You must have been drunk.’”*

Fear was especially heightened in cases where someone had been involved in a fight, or the injury was a result of domestic violence. In these instances, participants reported that they would not present at a health service at all, or if they did, would try and leave as soon as possible.

Linda (F, >45): *“No woman will wanna go to the hospital. Not one. Because that's her man. And she knows if she goes to the hospital, the police are going to be involved with it. And then family and children service … and there's a whole range of people who will be involved with that altercation. So she'd rather suffer than go to the hospital. If she does go to hospital, she will find a way of walking out or, um, not getting treated at all. Because she got abuse of the man still waiting for her. Because she's got to go back and care for her children.”*

Participants were more likely to present children for assessment at a health service, however, expressed a real fear that their children would be taken away from them.

Hannah (F): *“You don't want to take your kid [to the health service] … and then, you know your kid will be in danger.”*

Nell (F): *Yep.* *“My kid fell off the bed.”*

Hannah (F): *“And you get questions.”*

Nell (F): *“He had a slight blood nose from it. And I took him to the doctor … and they're like, ‘Oh, well, we're gonna have to report you.’ ‘Oh well I don't really want to ever bring him again! … If you're gonna take my baby away.’”*

## Discussion

This study examined the awareness and understanding of concussion in Aboriginal people with training in health or first response, living in Perth, Western Australia. It also explored the factors that may impact presentation for assessment after a potential concussion is sustained. While modes of sustaining a concussion were well understood, findings provide evidence that the concept of concussion as a specific diagnosis, rather than an all-encompassing term reflecting all types of traumatic brain injury is not well understood. Participants articulated key concepts – the complexity of concussion as a diagnosis, symptom minimization and lack of cultural safety within health services – as barriers to presenting for assessment after sustaining a potential concussion. They also reported concussion knowledge and awareness being acquired via word of mouth, lived experience including in community sports and via the media. Our findings contribute to a greater understanding of how concussion is viewed by Aboriginal peoples and highlight the need for co-designed solutions to improve understanding of concussion and optimize post-concussion care.

The lack of clarity around what constitutes a concussion and how this is differentiated from other traumatic brain injury diagnoses is likely to result in confusion relating to how, when and where it is most appropriate to seek assessment after a potential concussion is sustained. Such confusion is further complicated by concussion knowledge acquisition, even in this high health literacy group, having been via relatively low-detail information sources (lived experience or observation of sporting events). This highlights the need for information-rich, culturally appropriate and safe, concussion education which may include what symptoms and signs are red flags requiring ambulance/hospital attendance, what symptoms and signs suggest a diagnosis of concussion, and options regarding where to present for assessment. Emphasis on the fact that concussion does not require a loss of consciousness and information about its subtle signs is recommended [28], as is reinforcement of the fact that concussion can occur from a transmitted force to the brain from the body, not just a ‘head knock’. Such education must be community-led and co-designed and may be run through schools and other educational facilities, via local health services, sporting clubs or community centres, or on social media and other media platforms. In this high-health literacy group, further investigation of where they go to seek contemporary information on current practice is warranted. This may assist in providing a target for educational material relating to concussion for Indigenous Australians' working in healthcare and first response.

Given that attendance at community sporting and televised sports was commonly cited as places where awareness of and knowledge about concussion were gained, there may be a role for education through sport, with televised events being a potential avenue for ‘bite-sized’ inclusive educational snippets run during game breaks and commentary segments. The inclusion of information relating to the identification of red flags and when to call an ambulance may assist in the identification of alternative injury, and information regarding concussion symptoms, signs and relevant acute and subsequent management, as well as resource promotion, may be particularly useful inclusions.

Despite knowledge of the mode of concussion awareness, it was identified that appropriate action may not be taken upon receipt of a potential concussion. Co-designed, culturally appropriate health education is recommended to assist in the identification of symptoms and signs that suggest the need for immediate medical attention versus those requiring timely but non-immediate medical assessment, and how to access such care. Such education may include information that ensures adequate understanding and knowledge of concussion to reduce the likelihood of symptom minimization, as well as information relating to health services, their funding models and access.

While limited concussion knowledge does suggest a need for education, it has been shown in athletic populations that concussion education and prior concussion knowledge are not an effective deterrent for under-reporting [15]. This suggests lack of knowledge is only one factor playing a role in whether people present for concussion assessment. Sport has typically been a place where Indigenous Australians have felt some sense of belonging and acceptance, and this may explain why sporting culture and commitment to the team influence the trivialization of concussion symptoms and signs. This is supported by research in sporting populations where symptom minimization is identified [29] and the team is perceived as more important than a single individual [30].

Australia's suppressive history [31] of Indigenous Australians goes some way to explaining why health is not prioritized among this population with past experiences resulting in long-term trans-generational physical and psychological effects that adversely affect health-seeking behaviours and contribute to hospital avoidance [32]. Participants reported that particularly those from the Stolen Generation associate hospitals with disappearance and death [33]. This is further compounded by the low numbers of Indigenous Australians working in patient-facing roles in general hospitals and healthcare facilities [34]. Such factors play a role in Indigenous Australians not expecting to be treated with respect, or taken seriously, on presentation to health services and expecting that assessments and subsequent care will not be culturally appropriate resulting in inadequate assistance.

Given a participant reported perceived disingenuity of health services specifically funded by the Government for Indigenous peoples, it is clear that local Indigenous community engagement is crucial in the development of appropriate concussion care pathways, in all healthcare settings [35]. Increased numbers of Indigenous Australians within health services are expected to improve community engagement and health education related to concussion, as has been effective in diabetes care [36], as well as resulting in improved health and well-being [37]. Co-design of services has been effective in breaking down the barriers between service providers and the community [38] and the co-design of community-led education resources for Indigenous Australians to improve concussion literacy, but also to facilitate the understanding of the Australian healthcare system and Medicare, is a possible starting point. Providing information about career options and providing placements and financial, peer and mentor support to assist those wishing to pursue careers in healthcare and health education [39] is also recommended. Improvement in Indigenous male representation in the healthcare workforce may also assist in increasing health-seeking behavior in Indigenous men, as has been previously demonstrated in the Northern Territory [40].

Cultural competency in all settings from initial contact to discharge remains a must for non-Indigenous healthcare workers. A local community approach is suggested where Indigenous Elders are included on the boards of local hospitals and support Primary Health Networks to ensure appropriate governance, with medical/healthcare practices subject to the provision of evidence of meaningful cultural competence for accreditation. Such processes in health are similar to those identified as key to working with Indigenous Australians in research [41]. Medical practitioners and allied health workers involved in the assessment and management of people presenting with concussion are likely to benefit from Indigenous Australian-led cultural awareness and safety training including regular contact with those with lived experience. Family supports are also critical, supporting those with carer's duties to seek assessment and allowing support from extended family. Measurement of cultural safety from Indigenous Australian patients' perspectives should be an accepted norm, with the use of objective validated performance measures [42].

Participants reported experiences of, or fear of, authorities being contacted on presentation to a health service, and this played a factor in whether they would present for assessment following a potential concussion injury. The rate of Indigenous Australian children in out-of-home care is 11-times the rate of non-Indigenous children [43] with this disparity starting at infancy [44]. Until this is addressed, there will continue to be Indigenous Australians who will choose not to present to health services for fear of prejudice. Literature suggests that allegations of child maltreatment are common in families where the mother has presented to the hospital following an assault [45], further reinforcing to Indigenous Australians that presentation to medical facilities is ‘unsafe’ and increases the risk of their child being removed. Such practices are traumatizing, particularly where a history of child removals has resulted in intergenerational trauma and are likely to increase the risk of children with a concussion not receiving post-injury assessment and best practice management.

### Strengths & limitations

To our knowledge, this research is the first to generate preliminary evidence identifying areas where education and support are required to improve the identification, assessment and management of concussion in Indigenous Australians and the barriers impeding presentation to a health service for assessment and management after sustaining a potential concussion. Our sample was a high health literacy group, and we are conducting further research with Indigenous Australians outside the health and first responder community to confirm if our findings are consistent.

A clear strength of this research is that data collection was led by an Aboriginal Elder, which both facilitated a culturally safe environment and assisted with the ongoing education and learning of non-Aboriginal investigators working with Aboriginal colleagues and study participants. Further, the yarning methodology facilitated rich data collection resulting in an improved understanding of this important area of research. Research findings will be fed back via the RTO and Aboriginal Elder (TH-W) to participants, and relevant health, community and Aboriginal Community Controlled Health Organizations. Incidental participant concussion education was provided, as well as sharing of pre-existing concussion consumer/patient resources.

There were also certain limitations. First, yarning sessions were of a mixed nature with two yarning circles and four 1:1 yarns being carried out. Difficulty linking yarning circle participants to their demographic data prevented further analysis relating to age, educational level, and the influence of family carer's responsibilities on participants' awareness and understanding of concussion. Second, one yarning circle was facilitated by non-Indigenous chief investigator GC which may have impacted the richness of the data collected. Third, formal data analysis was undertaken by non-Indigenous researcher KM. We acknowledge the difference in cultural positioning of people who are non-Indigenous which may have influenced the shaping of the research findings. Although there was no direct involvement of an Indigenous Australian in the formal data analysis (due to competing workloads), feedback and probing by the Aboriginal co-investigators during the iterative rounds of the preliminary analysis encouraged the consideration of alternative perspectives, meaning and issues. We trust the involvement and oversight of our Aboriginal colleagues during the data collection and analysis phases moderated said limitations.

## Conclusion

Concussion awareness and understanding among Aboriginal people with healthcare and first response backgrounds in Western Australia varied and typically resulted from lived experience or viewing of televised sports. Injury pathophysiology was poorly understood and while there was awareness of head injury identification and red flags, there was a lack of clarity when differentiating between concussion and other traumatic brain injuries. Barriers to seeking assessment and healthcare included limited concussion knowledge, the complexity of the condition and symptom minimization. Further barriers were identified as a lack of cultural competence within health services, including prejudice, and a paucity of Indigenous healthcare workers, particularly in the male workforce. Indigenous Australian-led and owned concussion education is the first step in increasing concussion awareness. Medical care must strive to achieve cultural competence, take into account historical trauma and previous experiences that shape if, where and when Indigenous Australians present for concussion assessment, and support these populations to remain engaged throughout management. It is hoped that this will go some way to optimising outcomes among Indigenous Australians post-concussion.

**Summary points**

- Aboriginal peoples with high health literacy, living in Perth, Western Australia engaged in research topic yarning relating to concussion awareness and knowledge.
- Participants had good understanding of how concussion injury can occur.
- Identification of concussion as a diagnosis is complex.
- Difficulty differentiating concussion from other conditions occurs.
- Symptom minimization is common.
- There are barriers to seeking healthcare after sustaining a potential concussion.
- Awareness and knowledge relating to concussion was gained from televised and community sport, media and word of mouth despite participants having health or first responder experience.
- There is a need for Aboriginal and Torres Strait Islander led and co-designed concussion education.
- Further research in Aboriginal and Torres Strait Islander peoples, especially those without high health literacy backgrounds, is needed to further identify areas where improvement is needed to optimize outcomes after concussion injury.

## Footnotes

1 The Noongar are Aboriginal Australian peoples who live in the south-west of Western Australia.

2 A word used by the Indigenous Australians of  the south-west corner of Western Australia (Noongar) to describe people of white/Caucasian decent.

3 Australian Government driven strategy endorsed in 2008 that aims to close the health and life expectancy gap between Aboriginal and Torres Strait Islander peoples and non-Aboriginal Australians.
